# TNKS1BP1 functions in DNA double-strand break repair though facilitating DNA-PKcs autophosphorylation dependent on PARP-1

**DOI:** 10.18632/oncotarget.3137

**Published:** 2015-02-04

**Authors:** Lian-Hong Zou, Zeng-Fu Shang, Wei Tan, Xiao-Dan Liu, Qin-Zhi Xu, Man Song, Yu Wang, Hua Guan, Shi-Meng Zhang, Lan Yu, Cai-Gao Zhong, Ping-Kun Zhou

**Affiliations:** ^1^ School of Public Heath, Central South University, Changsha, Hunan Province 410078, P. R. China; ^2^ Department of Radiation Toxicology and Oncology, Beijing Key Laboratory for Radiobiology, Beijing Institute of Radiation Medicine, Beijing 100850, P. R. China; ^3^ School of Radiation Medicine and Protection, Medical College of Soochow University, Collaborative Innovation Center of Radiation Medicine of Jiangsu Higher Education Institutions, Suzhou 215123, China; ^4^ Department of Radiation Oncology, University of Texas Southwestern Medical Center, Dallas, TX 75390, USA

**Keywords:** TNKS1BP1, radiation, DNA-PKcs, DNA repair, poly(ADP-ribosyl)ation

## Abstract

TNKS1BP1 was originally identified as an interaction protein of tankyrase 1, which belongs to the poly(ADP-ribose) polymerase (PARP) superfamily. PARP members play important roles for example in DNA repair, telomere stability and mitosis regulation. Although the TNKS1BP1 protein was considered to be a poly(ADP-ribosyl)ation acceptor of tankyrase 1, its function is still unknown. Here we firstly identified that TNKS1BP1 was up-regulated by ionizing radiation (IR) and the depletion of TNKS1BP1 significantly sensitized cancer cells to IR. Neutral comet assay, pulsed-field gel electrophoresis, and γH2AX foci analysis indicated that TNKS1BP1 is required for the efficient repair of DNA double-strand breaks (DSB). The TNKS1BP1 protein was demonstrated to interact with DNA-dependent protein kinase (DNA-PKcs) and poly(ADP-ribose) polymerase 1 (PARP-1), by co-immunoprecipitation analysis. Moreover, TNKS1BP1 was shown to promote the association of PARP-1 and DNA-PKcs. Overexpression of TNKS1BP1 induced the autophosphorylation of DNA-PKcs/Ser2056 in a PARP-1 dependent manner, which contributed to an increased capability of DNA DSB repair. Inhibition of PARP-1 blocked the TNKS1BP1-mediated DNA-PKcs autophosphorylation and attenuated the PARylation of DNA-PKcs. TNKS1BP1 is a newly described component of the DNA DSB repair machinery, which provides much more mechanistic evidence for the rationale of developing effective anticancer measures by targeting PARP-1 and DNA-PKcs.

## INTRODUCTION

DNA double-strand breakage (DSB) induced by either ionizing radiation or genotoxic chemicals is a particularly hazardous type of DNA damage to dividing cells, because it involves a break in both DNA strands nearby in the double helix. There are two major interconnected and collaborative pathways to repair this damage: homologous recombination (HR) and non-homologous end joining (NHEJ) [1]. Due to needing a homologous template to guide repair of the broken DNA strand, the HR repair pathway is believed to be activated after DNA replication in the late S and G2 phases of the cell cycle. Whereas NHEJ repair could mediate the direct re-ligation of the broken DNA molecule, this type of repair does not require a homologous template and is not restricted to a particular phase of the cell cycle. Thus, NHEJ is thought to be the prevailing pathway for DSB repair [2]. The DNA dependent protein kinase complex (DNA-PK) is a critical component of the NHEJ pathway, which consists of a Ku70/Ku80 heterodimer and the DNA dependent protein kinase catalytic subunit (DNA-PKcs). DNA-PK plays an essential role in the pathway of NHEJ repair, including initiating DSB recognition, regulating access to breaks, promoting repair machinery assembly and DNA molecule ligation [3, 4]. As the catalytic subunit, DNA-PKcs is a member of the phosphatidylinositol-3 (PI-3) kinase-like family (PI3KK), and it is recruited to DNA damage sites by the Ku70/Ku86 heterodimer [5, 6]. The activation of DNA-PKcs in response to DNA damage is dependent on its autophosphorylation within two cluster sites (ABCDE and PQR) [4, 7] and/or phosphorylated by ATM [8]. The activated DNA-PKcs can phosphorylate a series of canonical repair factors in the NHEJ pathway, including Ku70/Ku86, Artemis, XRCC4, DNA ligase IV, XLF, Werner (WRN) and other DNA damage response proteins: including polynucleotide kinase/phosphatase (PNKP), histone H2AX, P53, CHK2, AKT [3, 9]. However, how the recruitment of DNA-PKcs onto DNA lesion sites stimulates its activity, or how its autophosphorylation is initiated, has not been fully clarified. It is particularly worthwhile to identify the potential proteins or other types of post-translational modifications which may modulate the DNA-PKcs activity and hence play a role in DNA double-strand break repair.

Poly(ADP-ribosyl)ation (PARylation), which is catalyzed by Poly(ADP-ribose) polymerases (PARP), recently has become an increasingly interested subject regarding protein post-translational modifications in the research field of DNA damage response and cancer therapeutic target exploration [10, 11]. PARP transfers negatively charged ADP-ribose groups from donor NAD^+^ molecules onto their target proteins, a process named poly(ADP-ribosyl)ation. At least three PARP molecules, PARP-1, PARP-2 and PARP-3, have been identified to be activated dependently of DNA in the cellular response to DNA double-strand breaks [11]. PARP-1, which mediates about 85% of cellular poly-ADP-ribose (PAR) synthesis activity, plays a key role in DNA repair, including in HR, NHEJ, single-strand break repair and base excision repair [12]. Following DNA damage, PARP-1 quickly relocates to DNA damage sites and catalyzes protein PARylation [13, 14], which contributes to the recruitment of other DNA damage response factors onto DNA lesions [15–20]. Inhibition or depletion of PARP-1 leads to a DNA DSBs NHEJ repair defect in hamster and murine cells after ionizing radiation (IR), manifesting in prolonged existence of the phosphorylated form of γH2AX foci which represents the persistence of DSBs [21–23]. Previous studies showed that PARP-1 could interact with DNA-PKcs and Ku70/Ku80 heterodimer [23–25]. PARP-1 can be phosphorylated by DNA-PKcs, and in turn, PARP-1 ADP-ribosylates the DNA-PKcs and increases the activity of DNA-PKcs. Interestingly, PARP-1 mediated ADP-ribosylation and activation of DNA-PKcs does not rely on Ku70/86 heterodimers [24, 26, 27]. Hence, the molecular mechanism of how PARP-1 facilitates DNA-PKcs activation remains to be elucidated.

Tankyrase 1 (TNKS1), the TRF1-interacting ankyrin-related ADP-ribose polymerase, is a member of the PARP superfamily. Tankyrase 1 was found to play an important role in DNA repair, telomere length maintenance and mitotic progression regulation [28, 29], and has also been considered to be a potential target for cancer therapy [30, 31]. Previously, a tankyrase 1 binding protein 1 of 182 kDa (TNKS1BP1, also named TAB182) has been identified in the co-immunoprecipitates of Tankyrase 1 [32]. TNKS1BP1 resides in both the nucleus and the cytoplasm, and displays a complex pattern of subcellular localization with Tankyrase 1. TNKS1BP1 was assumed to be poly ADP-ribosylated by Tankyrase 1 [32, 33]. However, the biological function of TAB182 is still unknown. In this study, we report that the expression of TNKS1BP1 was increased by ionizing radiation and depletion of TNKS1BP1 significantly sensitized multiple cancer cell lines to γ-irradiation. Moreover, depletion of TNKS1BP1 significantly decreased the efficiency of repair of DNA double-strand breaks. TNKS1BP1 was found to interact directly with DNA-PKcs, Ku70, Ku86, and PARP-1. Overexpression of TNKS1BP1 promoted the interaction between PARP-1 and DNA-PKcs, and subsequently increased the phosphorylation of DNA-PKcs /ser2056. Furthermore, inhibition of PARP-1 activity blocked the TNKS1BP1 mediated DNA-PKcs activation. These results implied that TNKS1BP1 is a novel modulator of radiation-induced DNA-PKcs phosphorylation, and contributes to DNA double-strand break repair through facilitating PARP-1/DNA-PKcs interaction. Thus, the TNKS1BP1 may represent a therapeutic target to improve the effect of radiotherapy.

## RESULTS

### The contribution of TNKS1BP1 to the survival of irradiated cells

**Figure 1:**
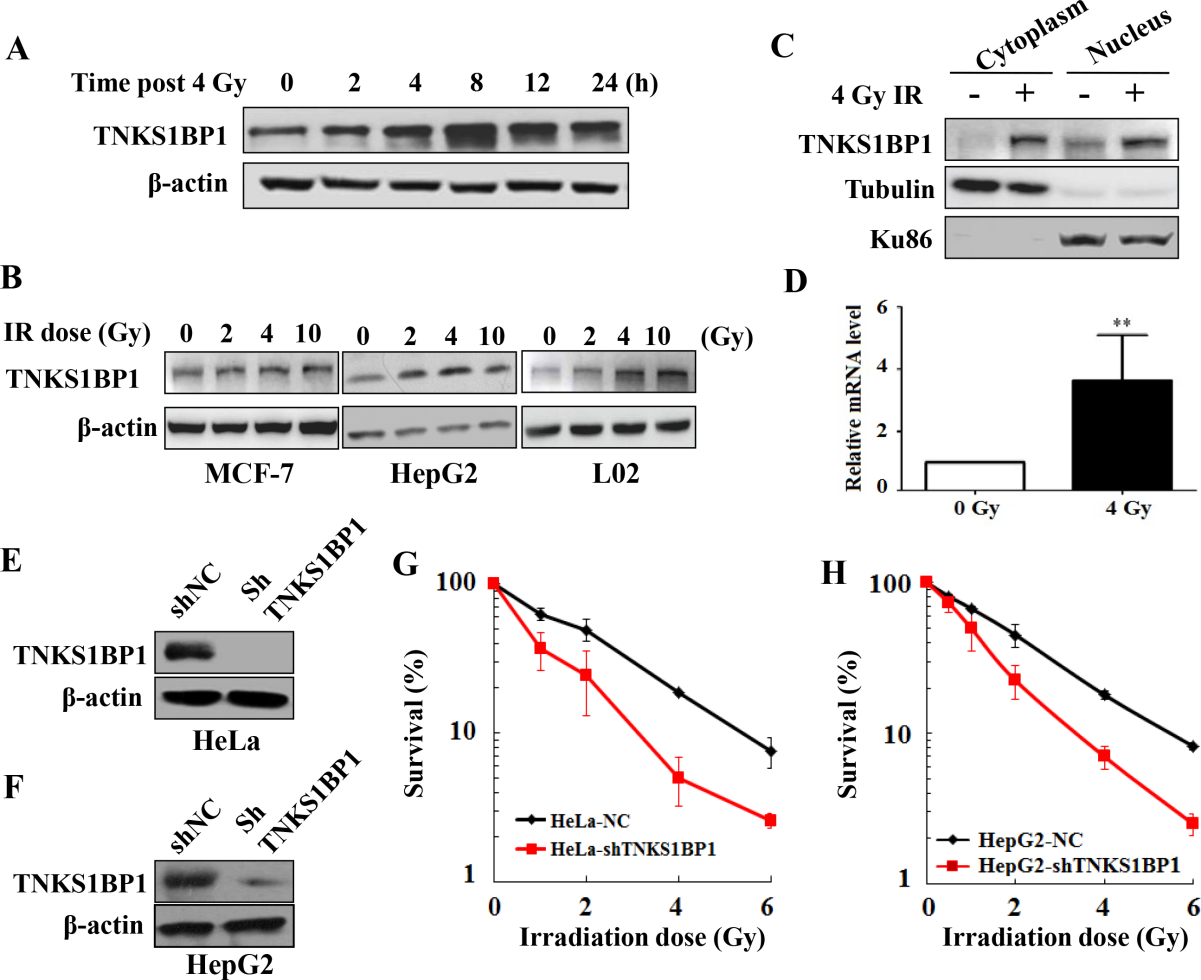
Upregulation of TNKS1BP1 by ionizing radiation (IR) and its effect on the radiosensitivity of cells **(A)** Immunoblotting hybridization showing the increased expression of TNKS1BP1 protein in HeLa cells at given times after exposed to 4 Gy of γ-rays. **(B)** Immunoblotting hybridization showing the increased expression of TNKS1BP1 protein in MCF7, HepG2 and L02 cells at 4 h after different doses of γ-rays. **(C)** Increased TNKS1BP1 protein levels in both cytoplasm and nuclei in HeLa cells at 4 h after 4 Gy of γ-rays. **(D)** Quantitative real-time RT-PCR analysis showing the increased mRNA expression of TNKS1BP1 in HeLa cells at 2 h after 4 Gy of γ-rays. **(E)** Depletion of TNKS1BP1 expression in HeLa cells mediated by specific shRNA. **(F)** Depletion of TNKS1BP1 expression in HepG2 cells mediated by specific shRNA. **(G)** TNKS1BP1 depleted HeLa-shTNKS1BP1 cells became much more sensitive to IR as compared to the control HeLa-NC cells. **(H)** TNKS1BP1 depleted HepG2-shTNKS1BP1 cells became much more sensitive to IR as compared to the control HepG2-NC cells.

We firstly investigated whether TNKS1BP1 was involved in the IR-induced DNA damage response. Growing HeLa cells were irradiated with 4 Gy γ-rays, and harvested at different time points post irradiation. Immunoblotting analysis revealed that the expression level of TNKS1BP1 increased at least as early as 2 h after irradiation. The inducible expression of TNKS1BP1 by ionizing irradiation reached a peak level at about 4 – 8 h (Figure 1A). To test whether the IR-induced expression occurred in different cell lines, we gave human breast cancer MCF-7 cells, human hepatocellular cancer HepG2 cells and human normal liver L02 cells different doses of γ-rays. Levels of TNKS1BP1 protein in cell lysates were detected by immunoblotting analysis at 4 h after irradiation. An increased expression of TNKS1BP1 was observed in these three cell lines after irradiation (Figure 1B). As TNKS1BP1 has been reported to localize in both cytoplasm and nucleus, we studied which part mainly contributes to IR-induced TNKS1BP1 overexpression by fractionating nucleus and cytoplasm and detecting the respective expression levels of TNKS1BP1. As shown in Figure 1C, the expression of TNKS1BP1 was increased in both the cytoplasm and nuclear fractions. We further performed a real-time PCR experiment and found that the mRNA levels of *TNKS1BP*1 were significantly increased in 4 Gy γ-irradiated HeLa cells (Figure 1D).

As the expression of TNKS1BP1 was upregulated in the irradiated cells, we further evaluated the role of TNKS1BP1 in determining the cellular radiosensitivity. For the above purpose, we established the *TNKS1BP*1 stably-knocked-down cell lines HeLa-shTNKS1BP1 (Figure 1E) and HepG2-shTNKS1BP1 (Figure 1F) through sustained expression of shRNA against *TNKS1BP*1 in HeLa cells and HepG2 cells, respectively. As shown in Figure 1G and 1H, knockdown of TNKS1BP1 markedly decreased the survival rates of both HeLa cells (Figure 1G) and HepG2 cells (Figure 1H) when compared to the control HeLa-NC and HepG2-NC cells after IR, respectively. These results demonstrated that TNKS1BP1 plays an important role in maintaining the surviving capability of cells against IR.

### TNKS1BP1 plays a role in DNA double-strand break repair

**Figure 2:**
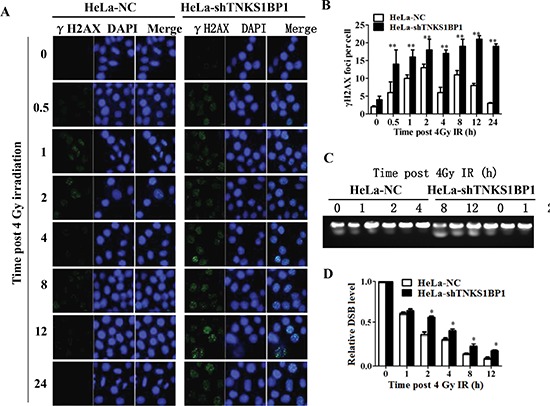
Depletion of TNKS1BP1 leads to defective DNA double-strand break repair **(A)** Immunofluorescence staining image of γH2AX foci in TNKS1BP1 depleted HeLa-shTNKS1BP1 cells and control HeLa-NC cells at given times after 4 Gy of γ-rays. **(B)** Residual number of γH2AX foci per cell after certain times of repair in 4 Gy irradiated cells. **P < 0.01. **(C)** Pulsed-gel electrophoresis (PFGE) pattern of DNA double-strand breaks and repair in 4 Gy-irradiated cells. **(D)** Repair kinetics of 4 Gy-induced DSBs detected by PFGE. *P < 0.05.

Decreased DNA DSB repair is an important mechanism by which cells become more sensitive to IR. To measure the repair kinetics of IR-induced DSBs, HeLa-NC and HeLa-shTNKS1BP1 cells were irradiated with 4Gy and harvested at the indicated time points. The Immunofluorescence analysis showed that γH2AX foci after irradiation in TNKS1BP1 knockdown HeLa cells exhibited prolonged repair kinetics as compared to control cells (Figure 2A and 2B). To further confirm this defect in DSB repair capacity of HeLa-shTNKS1BP1 cells, the pulsed-field gel electrophoresis assay (Figure 2C and 2D) and neutral comet assay (Supplementary Figure 1A and 1B) were performed to assess the repair efficiency of IR-induced DSBs. The results clearly indicated that the repair efficiency of IR-induced DSBs was significantly decreased in TNKS1BP1-suppresed HeLa cells.

The alteration of cell cycle progression is another critical cellular response to the DNA damage signal induced by IR. As shown in Supplementary Figure 2A and 2B, at 12 h post 4 Gy IR, HeLa-shTNKS1BP1 and HeLa-NC cells were largely arrested at G_2_/M phase. At 24 h after 4 Gy IR, HeLa-NC cells almost completely recovered from the G_2_/M blockage. However, HeLa-shTNKS1BP1 cells still exhibited robust G_2_/M arrest (50.6%) at the same time point. Furthermore, we investigated whether the shRNA-resistant TNKS1BP1 expressing vector could rescue this abnormal cell cycle progression phenotype in HeLa-shTNKS1BP1 cells. We found that the delayed recovery of G_2_/M arrest in HeLa-shTNKS1BP1 cells was rescued by stably overexpressing the shRNA-resistant Myc-TNKS1BP1 (Supplementary Figure 2C and 2D).

### TNKS1BP1 facilitates the phosphorylation of DNA-PKcs at Ser2056

**Figure 3:**
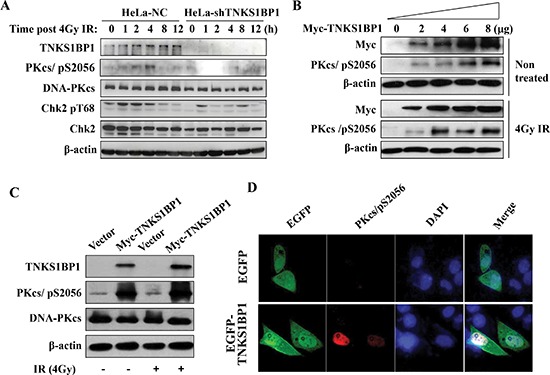
TNKS1BP1-medated autophosphorylation of DNA-PKcs/S2056 **(A)** Immunoblotting hybridization showing that depletion of TNKS1BP1 blocked the autophosphorylation of DNA-PKcs/S2056 and phosphorylation of CHK2/T68 induced by IR in HeLa cells. **(B)** Overexpression of TNKS1BP1 induced the autophosphorylation of DNA-PKcs/S2056 in HEK-293 cells either with or without irradiation. **(C)** shRNA-resistant Myc-TNKS1BP1 increased the autophosphorylation level of DNA-PKcs/S2056 in TNKS1BP1-depleted HeLa-shTNKS1BP1 cells. **(D)** Immunofluorescence image showing the autophosphrylation of DNA-PKcs/S2056 in HeLa cells transfected and expressing EGFP-tagged TNKS1BP1 plasmid.

Since TNKS1BP1 is involved in DNA damage response, and plays a role in DNA DSB repair, we investigated whether the depletion of TNKS1BP1 in HeLa cells would disturb the signaling transduction of the DNA damage response induced by IR. The Ser2056 has been considered as an autophosphorylation site of DNA-PKcs, and its phosphorylation represents the activation of DNA-PKcs. Our result indicated that the phosphorylation of DNA-PKcs/Ser2056 was significantly impaired in TNKS1BP1-deficient HeLa cells (Figure 3A). DNA-PKcs has been previously shown to phosphorylate Chk2 in response to DNA damage induced by IR or during normal mitosis progression [34–36]. As shown in Figure 3A, the IR-induced phosphorylation of Chk2 at T68 site was much weaker in TNKS1BP1 deficient HeLa cells compared to control cells, indicating that the deficiency of TNKS1BP1 disturbed the activation of the down-stream substrates of DNA-PKcs. Moreover, overexpression of TNKS1BP1 increased the phosphorylation of DNA-PKcs/Ser2056 with or without the IR-induced DNA damage (Figure 3B). More importantly, we transfected the shRNA-resistant Myc-TNKS1BP1 expressing vector into TNKS1BP1 knockdown HeLa-shTNKS1BP1 cells, and found that the phosphorylation level of DNA-PKcs/Ser 2056 was restored (Figure 3C). To further confirm the contribution of TNKS1BP1 to the phosphorylation of DNA-PKcs/Ser2056, we performed the immunofluorescence assay. HeLa cells were transiently transfected with pEGFP-C1-TNKS1BP1, or pEGFP-C1 for the control, for 36 h, and then subjected to immunofluorescence analysis. As shown in Figure 3D, when the cells were transfected and expressing TNKS1BP1, a significantly increased level of DNA-PKcs/pSer2056 was displayed. Taken together, our results demonstrated that TNKS1BP1 facilitated the phosphorylation of DNA-PKcs at the Ser2056 site.

### TNKS1BP1 mediates the interaction of DNA-PKcs and PARP-1

**Figure 4:**
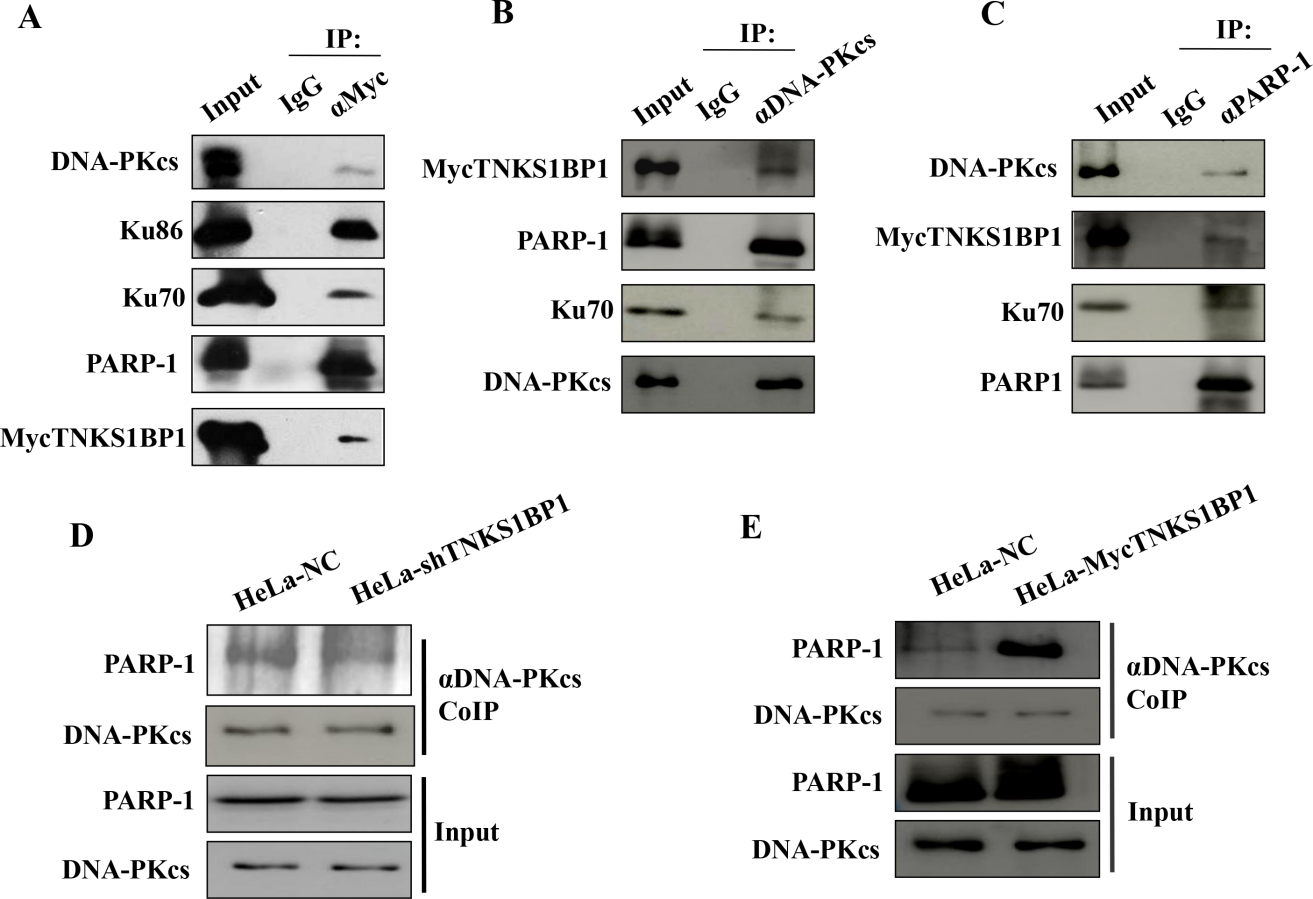
TNKS1BP1 mediated the interaction of DNA-PKcs and PARP-1 **(A)** Interaction of TNKS1BP1 with the DNA-PK complex and PARP-1 was shown by co-immunoprecipitation (Co-IP) with antibody against myc from HeLa cells transfected with myc-tagged TNKS1BP1, or with IgG control. **(B)** Interaction of DNA-PKcs with TNKS1BP1 and PARP-1 was shown by Co-IP with the antibody against DNA-PKcs from HeLa cells transfected with myc-tagged TNKS1BP1, or with IgG control. **(C)** Interaction of PARP-1 with TNKS1BP1 and DNA-PKcs was shown by Co-IP with the antibody against PARP-1 from HeLa cells transfected with myc-tagged TNKS1BP1, or with IgG control. **(D)** A decreased PARP-1 level in the co-immunoprecipitated complex of the antibody against DNA-PKcs in TNKS1BP1-depleted HeLa-shTNKS1BP1 cells as compared to that from control HeLa-NC cells. **(E)** Increased PARP-1 level in the co-immunoprecipitated complex of the antibody against DNA-PKcs in TNKS1BP1-overexpressed HeLa-mycTNKS1BP1 cells as compared to that from control HeLa-NC cells.

To clarify whether TNKS1BP1 interacts with DNA-PKcs, the specific antibody against TNKS1BP1/TAB182 was used to immunoprecipitate the TNKS1BP1-interacting complex from HeLa cell extracts. Western blotting analysis demonstrated that the DNA-PK complex (Ku70/Ku80/DNA-PKcs) was detected in the immunoprecitates of TNKS1BP1 (Figure 4A). In addition, PARP-1 was also shown in the TNKS1BP1-interacted complex. Moreover, TNKS1BP1 and PARP-1 were detected in the complex immunoprecipitated by the antibody against DNA-PKcs (Figure 4B), and DNA-PKcs and TNKS1BP1 were found in the complex immunoprecipated by the antibody against PARP-1 (Figure 4C). Therefore, DNA-PK, TNKS1BP1 and PARP-1 could form a multiple complex in the DNA damage response.

The interaction between PARP-1 and DNA-PK has also been previously described in several independent studies [23–25]. In order to understand the effect of TNKS1BP1 on the interaction of DNA-PKcs and PARP-1, we used the DNA-PKcs antibody to perform the immunoprecipitation assays from lysates of the cells with a control normal level of TNKS1BP1 (HeLa-NC), shRNA-mediated depleted TNKS1BP1 (HeLa-shTNKS1BP1), and overexpressed TNKS1BP1 (HeLa-mycTNKS1BP1). As shown in Figure 4E, the association of PARP-1 with DNA-PKcs was weakened in HeLa-shTNKS1BP1 cells. Whereas, overexpressed TNKS1BP1did promote the association of PARP-1 with DNA-PKcs (Figure 4E). Therefore, TNKS1BP1 could play a role in mediating the association of DNA-PKcs and PARP-1.

### Activation of DNA-PKcs mediated by TNKS1BP1 relies on the ADP-ribose polymerase activity of PARP-1

**Figure 5:**
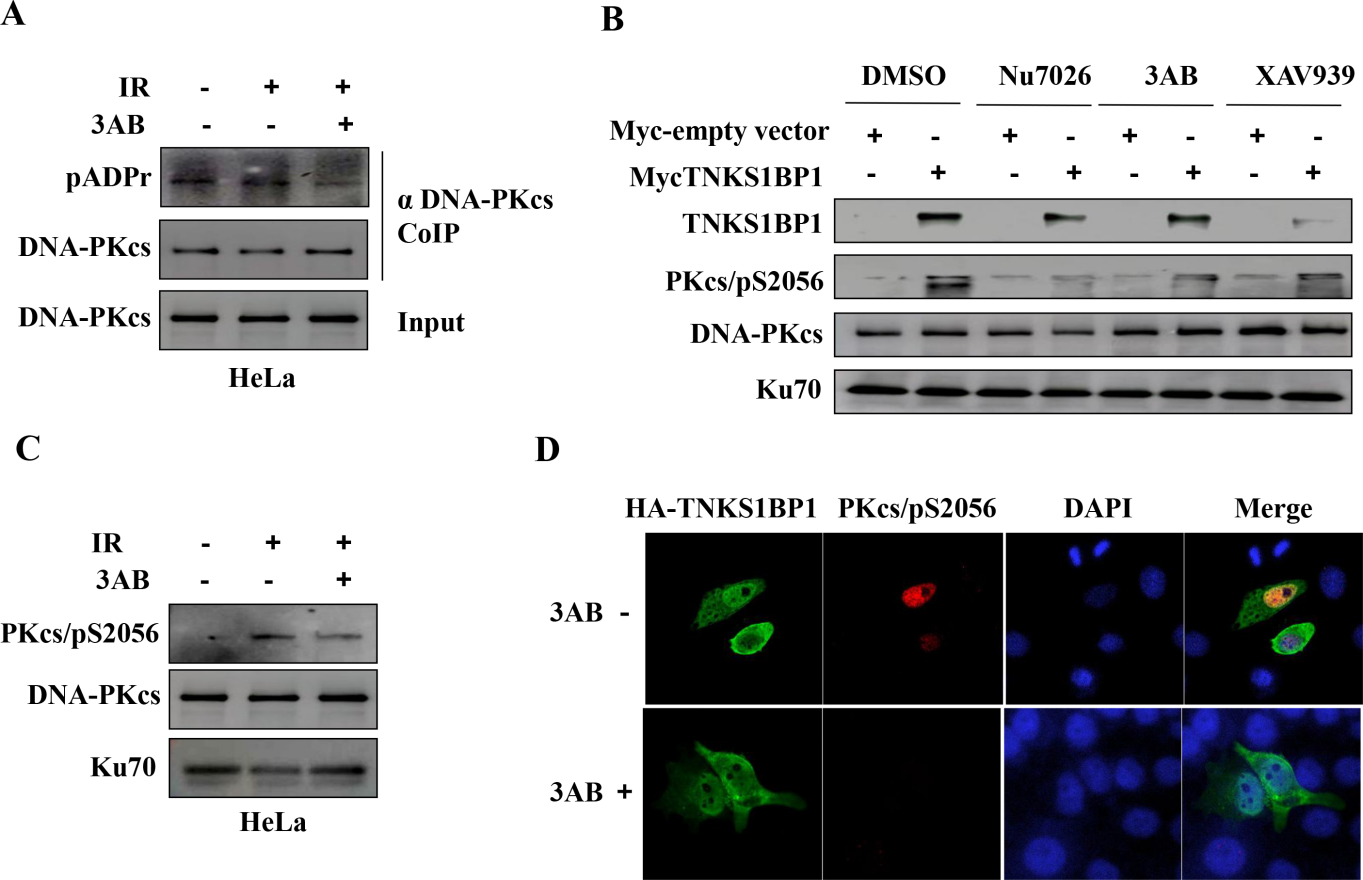
PARP-1 is responsible for TNKS1BP1-induced autophosphorlation of DNA-PKcs/S2056 **(A)** PARP-1 inhibitor 3-AB decreased the poly(ADP-ribosy)lation of DNA-PKcs. The Co-IP complex of the antibody against DNA-PKcs was separated by SDS-PAGE and immunoblotted with anti-pADPr antibody, anti-DNA-PKcs antibody. The immunoblotting of the input of cell lysates was used as sample control. **(B)** TNKS1BP1-induced autophosphorylation of DNA-PKcs/S2056 was attenuated by DNA-PKcs inhibitor Nu7056, PARP-1 inhibitor 3AB. The action of tankyrase 1 inhibitor XAV939 was relative weak. **(C)** PARP-1 inhibitor 3-AB decreased the autophosphorylation of DNA-PKcs/S2056. **(D)** Immunofluorescence image showing that the TNKS1BP1-induced autophosphrylation of DNA-PKcs/S2056 was blocked by 3-AB. HEK-293 cells were transfected with plasmid of HA-tagged TNKS1BP1, and subjected to co-immunofluorescence staining with DNA-PKcs/pS2056 antibody and HA antibody 48 h after plasmid transfection.

PARP-1 has been suggested to PARylate DNA-PKcs, and thereby to stimulate the kinase activity of DNA-PKcs [19, 21, 23, 24]. Here we also observed the PARylation modification of DNA-PKcs in the cells, which was attenuated by the PARP-1 inhibitor 3-AB (Figure 5A). To test whether TNKS1BP1-mediated activation of DNA-PKcs relies on the activity of PARP-1, we treated TNSKS1BP1 overexpressed HeLa-mycTNKS1BP1 cells and control HeLa-NC cells with an inhibitor of DNA-PKcs (Nu7026), or an inhibitor of PARP-1 (3-AB), and an inhibitor of Tankyrase 1 (TNKS1) (XAV939), and used DMSO as control. As shown in Figure 5B, the phosphorylation of DNA-PKcs/S2056 was largely blocked by Nu7026. The PARP-1 inhibitor 3-AB partially attenuated the TNKS1BP1-mediated DNA-PKcs phosphorylation at Ser2056. However, XAV939 displayed a relative weaker role as compared to the effect of 3-AB (Figure 5B). Moreover, phosphorylation of DNA-PKcs induced by ionizing radiation was also attenuated by the PARP-1 inhibitor 3-AB (Figure 5C). To further confirm the effect of PARP-1 on TNKS1BP1-mediated DNA-PKcs phosphorylation, we performed the immunofluorescence assay. There was an increased phosphorylation of DNA-PKcs mediated by overexpressing HA-tagged TNKS1BP1, which was largely blocked by PARP inhibitor 3-AB (Figure 5D). Taken together, these results demonstrated that TNKS1BP1 mediated the phosphorylation of DNA-PKcs at Ser2056,which could be attenuated by PARP inhibitors.

## DISCUSSION

TNKS1BP1 was firstly identified as an interaction partner of tankyrase 1, and it can be poly(ADP-ribosyl)yated by tankyrase 1 *in vitro* and *in vivo* [32, 33]. Here, we demonstrated that TNKS1BP1 is involved in the IR-induced DNA damage response. An increased expression of TNKS1BP1 was firstly observed in the cells after irradiation. Depletion of TNKS1BP1 impaired the efficiency of DNA double-strand break repair and significantly increased the sensitivity of cells to IR. TNKS1BP1-deficient HeLa displayed a much higher level of residual γH2AX foci, a DSB biomarker, after certain times of post-irradiation repair as compared to TNKS1BP1-proficient HeLa cells, consistent with prolonged γH2AX kinetics. The defective DSB repair caused by depletion of TNKS1BP1 was also confirmed using comet and neutral pulsed-field gel electrophoresis assays. It has been reported that the localization of TNKS1BP1 in cells exhibits a heterochromatic pattern in the nucleus. The large internal acidic region (pI = 4.3) of TNKS1BP1 contributes an acidic protein with pI = 4.6, which may facilitate its association with chromatin through binding to basic chromatin proteins, such as histones [32]. Previous observations suggested that TNKS1BP1 associated with heterochromatin protein 1 (HP1) [32]. Therefore, we assumed that as a chromatin associating protein, TNKS1BP1 may play a role in the assembly of DNA damage response protein complex around the DNA damage site when DNA DSBs occur.

In response to DSBs, DNA-PKcs is phosphorylated at multiple sites, among which Ser2056 is a bona fide autophosphorylation site. The autophosphorylation of DNA-PKcs/S2056 can be induced by ionizing radiation and other DNA damage agents [4, 7, 37], and is necessary for the activation of the DSB NHEJ repair pathway. Our study demonstrated that overexpression of TNKS1BP1 effectively induced the autophosphorylation of DNA-PKcs/S2056. This TNKS1BP1-related autophosphorylation of DNA-PKcs was abolished by its kinase inhibitor Nu7026. However, TNKS1BP1 did not induce the phosphorylation of DNA-PKcs at the site of T2609 (data not shown). It appears that TNKS1BP1 plays a role in activating DNA-PKcs through regulating its autophosphorylation.

In order to reveal the mechanism of TNKS1BP1 in regulating DNA-PKcs autophosphorylation, we identified the interacting proteins by co-immunoprecipitation assays using the antibody against TNKS1BP1/TAB182. Our study demonstrated that TNKS1BP1 interacted with the DNA-PKcs/Ku70/Ku86 complex and PARP-1. Although it has previously been reported that DNA-PKcs and PARP-1 are substrates for each other [24, 27], the purified DNA-PKcs and PARP-1 did not interact directly *in vitro* [24]. Therefore, their interaction in cells is believed to be mediated by other protein(s). Our study showed that depletion of TNKS1BP1 attenuated the association of DNA-PKcs and PARP-1, implying that TNKS1BP1 is responsible for mediating the interaction of DNA-PKcs and PARP-1.

PARP-1 is the most abundant member of the poly(ADP-ribose) polymerase superfamily in cells. PARP-1 has a carboxyl-terminal catalytic domain that polymerizes linear or branched chains of ADP-ribose (ADPR) from donor nicotinamide adenine dinucleotide (NAD^+^) onto the target proteins. Poly(ADP-ribosyl)ation of proteins, also called PARylation, by PARP-1 is one type of post-translation modifications, which is involved in regulating gene expression and DNA damage repair. It has been reported that DNA-PKcs was PARylated by PARP-1 in HeLa cells after the stimulation of IFN-γ [25], and this PARylation is required for the activation of DNA-PKcs. Here, we also detected the PARylation of DNA-PKcs, which was abolished by the PARP-1 inhibitor 3-AB. Similar to DNA-PKcs, PARP-1 can be activated by DNA strand breaks and is also a key component involved in DNA damage recognition, repair and signal transduction. Accumulated evidence suggested that PARP-1 and DNA-PKcs cooperated within the same pathway to promote DSBs repair. Inhibition of PARP-1 by a specific inhibitor not only impaired the efficiency of single strand break repair and base excision repair, but also decreased the efficiency of the NHEJ pathway of DSB repair when cells were exposed to ionizing radiation [21, 26]. Several PARP-1 inhibitors have been explored to cancer therapy either alone or in combination with radiotherapy or other DNA-damaging drugs [38, 39]. In addition, DNA-PKcs was shown overexpressed or highly activated in many tumors and its upregulation was correlated with advanced stage and poor patient prognosis [40–42]. A series of studies have demonstrated that targeting DNA-PKcs is an attractive strategy of cancer therapy [35, 42–44]. Recently, Verghese et al reported that the miR-26b was a highly deregulated microRNA in the carcinoma-associated fibroblasts (CAFs) in breast cancers, and the reduced miR-26b expression caused an enhancement of the fibroblast migration and invasion. *TNSK1BP1* was identified to be one of the direct downstream key targets of miR-26b. Moreover, the high expression of *TNSK1BP1* in cancer stroma was significantly associated with the increased rates of breast cancer recurrence [45]. Therefore, all of these three molecules DNA-PKcs, PARP-1 and TNSK1BP1 are obviously implicated whether with the cancers prognosis or with the outcome of radio- or chemotherapy. Our results has provided novel information demonstrating that TNKS1BP1 functions as a bridge linking the PARP-1 and DNA-PKcs, a critical component of the NHEJ pathway of DNA DSB repair, to stimulate the autophosphorylation of DNA-PKcs in the cellular response to ionizing radiation-induced DNA double-strand breaks.

In summary, for the first time we demonstrated the biological function of TNSK1BP1 participating in the regulation of DNA DSB repair. TNKS1BP1 mediates the interaction of DNA-PKcs and PARP-1, which leads to activation of DNA-PKcs autophosphorylation. The PARylation of DNA-PKcs by PARP-1 appears to play a role in above process.

## MATERIALS AND METHODS

### Plasmids, antibodies and reagents

TNKS1BP1 /TAB182 expression vector (PLPC-MYC-TAB182) was kindly provided by Dr. Susan Smith (Kimmel Center for Biology and Medicine of the Skirball Institute, New York University School of Medicine). PCMV-HA-TNKS1BP1 vector was generated by polymerase chain reaction (PCR) cloning. shRNA-TNKS1BP1-U6/Hygromycin vectors and shRNA-NC-U6/hygromycin vectors were purchased from Genepharma. To obtain the RNA interference resistant TNKS1BP1 expression plasmid (PCMV-HA-shiTNKS1BP1), the region targeted by the TNKS1BP1 shRNA was changed to GGAGAGTTTCTCAAGTCGAGGGAGCGTGGA. Mutagenesis of TNKS1BP1 was done using the Q5 hot start High-fidelity DNA polymerase. DNA-PKcs Inhibitor (Nu7026), PARP inhibitors (3-AB, XAV939), Propidium Iodide (PI) and DAPI were purchased from Sigma. All antibodies were commercial products. Anti-actin, anti-DNA-PKcs, anti-Ku86, anti-PARP-1, anti-TAB182 (TNKS1BP1), anti-Myc (9E10) and anti-HA were purchased from Santa Cruz. Anti-Ku70, anti-pADPr and anti-phospho-DNA-PKcs (Ser2056) were purchased from Abcam. Anti-Phospho-H2AX (Ser139) was purchased from Millipore, AlexaFlour 568-goat anti-rabbit IgG and AlexaFlour 488-goat anti-mouse IgG were purchased from Invitrogen.

### Cell lines, culture and ionizing radiation

All cell lines were obtained from ATCC. HeLa human cervical cancer cell line, HepG2 human hepatocellular cancer cell line, MCF-7 human breast cancer cell line and human embryonic kidney HEK 293 cells were cultured in DMEM medium (Gibco) supplemented with 10% FBS (Hygclone), and maintained in a humidified incubator with 5% CO_2_, 37°C. HeLa-shTNKS1BP1 and HeLa-NC, HepG2-shTNKS1BP1 and HepG2-NC cells were generated from HeLa cells and HepG2 cells, respectively, via stably transfecting with shRNA-TNKS1BP1-U6/Hygromycin vectors targeting the TNKS1BP1 or a control shRNA-NC-U6/hygromycin vector. A cell line with stable overexpression of TNKS1BP1 was generated by transfecting PLPC-MYC-TNKS1BP1 or an empty vector (as a control) into HeLa cells. Colonies showing resistance to puromycin (2-ug/ml) were isolated and analyzed by immunoblotting. Plasmid transfection of HEK-293 cells was performed with Lipofectamine 2000 (Invitrogen) and the transfected HEK-293 cells were analyzed after 48 h. Cells were irradiated using ^60^Co γ-rays at a dose rate of 1.98 Gy/min.

### Immunoblotting hybridization and immunofluorescent staining

For immunoblotting (western blotting) analysis, cells with or without transfection of PLPC-MYC-TNKS1BP1, PCMV-HA-TNKS1BP1 or PCMV-HA-shiTNKS1BP1 plasmids for 48 h, were given 4Gy irradiation, and harvested at the indicated time points, and then lysed in the NETN lysis buffer (100 mM NaCl, 20 mM Tris-Cl (pH 8.0), 0.5 mM EDTA, 0.5% (v/v) Nonidet P-40 (NP-40)) containing a cocktail of protease inhibitors (Roche). The nuclear and cytoplasmic extracts were prepared from HeLa cells given 4Gy or no irradiation, using a NE-PER kit (Thermo Fisher Scientific). Equal amounts of protein from each sample were injected into SDS-PAGE, then separated and transferred onto a Nitrocellulose membrane (Millipore). The blotting was blocked with 5% milk power in TBST (20 mM Tris-HCl, 500 mM NaCl (pH7.5), 0.1% (v/v) Tween 20) for 1 h at room temperature, then incubated overnight with the indicated antibody and washed with TBST. Bands were visualized by Imagequant LAS500 (GE).

For immunofluorescent staining, HeLa, HeLa-shTNKS1BP1 or HeLa-NC were transfected with TNKS1BP1 expression vector for 48 h, and irradiated with a dose of 4 Gy, then fixed in chilled phosphate-buffer saline (PBS) containing 4% paraformaldehyde overnight at 4°C. The fixed cells were permeated in PBS containing 0.25% Trion X-100 for 30 min and washed with PBS for 3 times at room temperature, then blocked with 1% BSA in PBS for 30 min. The cells were incubated with the indicated primary antibodies for 12 h at 4°C, washed with PBS for 3 times, incubated with AlexaFlour 568-goat anti-rabbit IgG and AlexaFlour 488-goat anti-mouse IgG for 1 h at room temperature, washed with PBS for 3 times again, stained with DAP1 to visualize the DNA at room temperature, and observed using a LSM 510 laser-scanning confocal microscope (Zeiss).

### RNA isolation and *TNKS1BP1* mRNA expression analysis by quantitative real-time RT-PCR

Total RNA was extracted from HeLa cells harvested at 2 h post 4 Gy using TRlzol reagent (Invitrogen) as recommended by the manufacturer. 1 μg of total RNA was then used to reverse transcription using the ReverTra Ace qPCR RT Master Mix kit (Toyobo Life Science). Real-time PCR was performed for detecting *TNKS1BP1* expression with a Bio-Rad iCycler & iQ Real-time PCR systems (Bio-Rad) and a fluorescence-labeled SYBR Green real master Mix kit (TIANGEN Biotech (Beijing) Co. LTD.). Actin was used as an endogenous control. The sequence of forward and reverse primers for *TNKS1BP*1 and *actin* are the following: *TNKS1BP1* forward 5ʹ-GTCAGGACTTCTCCTTCATTGAG-3ʹ and reverse 5ʹ-AGGCCAGGAAAGAGGTTGACTT-3ʹ; *actin* forward 5ʹ-CAGAGCAAGAGAGGCATCCT-3ʹ and reverse 5ʹ-TTGAAGGTCTCAAACATGAT-3ʹ. Each sample was tested three times, and the expression levels of *TNKS1BP1* were normalized to actin mRNA.

### Immunoprecipitation assay

To detect the interaction complex of TNKS1BP1, DNA-PKcs, Ku and PARP-1, cells were lysed in a cold NTEN buffer (100 mM NaCl, 20 mM Tris-Cl (pH 8.0), 0.5 mM EDTA, 0.5% (v/v) Nonidet P-40 (NP-40)) for 20 min on ice, then the lysates were centrifugated at 12000 g for 30 min at 4°C. For immunoprecipitation, the supernatants were collected and mixed with 2 μg of the indicated primary antibodies, and incubated for 12 h at 4°C, then incubated with protein A/G agarose (Santa Cruz) for 4 h at 4°C. Immunoprecipitated complexes were collected by centrifugation at 1000 g for 3 min at 4°C, washed 4 times with NTEN buffer, and analyzed by western blotting hybridization.

### Colony formation assay

Exponentially growing cells were collected and counted. Then the cells were irradiated at the different doses of 0, 1, 2, 4, 6 Gy and plated into 60 mm dishes with a certain number of cells per dish, respectively. Cells were fed with DMEM medium supplemented with 10% FBS for 2 weeks, then fixed with 70% ethanol, and stained with 1% crystal violet. The number of colonies containing more than 50 cells was calculated on each dish.

### Pulsed field gel electrophoresis (PFGE)

For pulsed field gel electrophoresis (PFGE), after 20 Gy irradiation Hela-NC and HeLa-shTNKS1BP1 cells were harvested at the indicated time points, washed 3 times with PBS, resuspended with 50 μl cell resuspension buffer (10 mM Tris, pH 7.2, 20 mM NaCl, 50 mM EDTA). Then the cells were maintained at 50°C and mixed with 2% LMP (50°C) at a ratio of 1:1 (v/v). After being placed into a gel pore, the cell-agarose mixture was maintained at 4°C for solidifying, and digested in proteinase K reaction buffer (100 mM EDTA, pH 8.0, 0.2% sodium deoxycholate, 1% sodium lauroyl sarcosine, 0.5 mg/ml proteinase K) at 50°C for 24 h. Then the cells/agarose mixture was washed 3 times in wash buffer (20 mM Tris, pH 8.0, 50 mM EDTA) gently at room temperature, and inserted into a 10% agarose gel and subjected to electrophoresis for 72 h under the conditions of 1.5 v/cm, 120 degree of angle, 16°C of circulating buffer temperature. Thereafter the agarose gel was visualized using a gel imaging system and analyzed by Quantity One Soft.

## SUPPLEMENTARY MATERIALS AND METHODS


