# Supplementary material for: TNKS1BP1 functions in DNA double-strand break repair though facilitating DNA-PKcs autophosphorylation dependent on PARP-1
Source: Oncotarget. 2015 Feb 4;6(9):7011–22. doi: 10.18632/oncotarget.3137 (PMC4466666; doi:10.18632/oncotarget.3137)
Supplement: Supplementary file 1 [file oncotarget-06-7011-s001.pdf]

SUPPLEMENTARY MATERIALS AND METHODS

Cell cycle analysis

Cells were given 4 Gy ionizing radiation, and harvested at the indicated time points, washed with PBS three times, then fixed in cold 70% ethanol (−20°C) overnight. The cell sediment was collected by centrifugation at 1000 g for 3 min, and washed 3 times with PBS, digested with 40 µl RNase A (10 mg/ml) for 30 min at 37°C, and stained with 10 µg/ml Propidium iodide for 15 min at room temperature. Cell cycle distributions were analyzed using flow cytometry. All experiments were repeated 3 times.

Comet assay

For the neutral comet assay, after being given 4Gy IR, the cells were collected at 0, 1, 2, 4 h after irradiation, and mixed with 0.625% low melting point agarose (LMP) at 37°C. Then the mixture was placed onto the top of a previously formed layer of 1% normal melting point agarose (NMP) on one slide, covered with a cover slip and placed at 4°C for 15 min. Then the cover slip was gently removed and the slide was placed in cold neutral lysis solution (2.5 M NaCl, 100 mM EDTA, 10 mM Tris base, 1% Triton X-100, 10% DMSO, pH = 8.0) for 3 h. The slide was washed 3 times in chilled neutralization buffer (90 mM Tris base, 90 mM boric acid, 2 mM Na_2EDTA, pH 8.0) for 1 h each time, and subjected to electrophoresis at 4°C for 25 min at 20 V. Thereafter, the slides were gently washed with PBS, and stained with 1 µg/ml PI for 5 min at room temperature. Cells were visualized at 200 × magnification using a fluorescence microscope.

SUPPLEMENTARY FIGURES

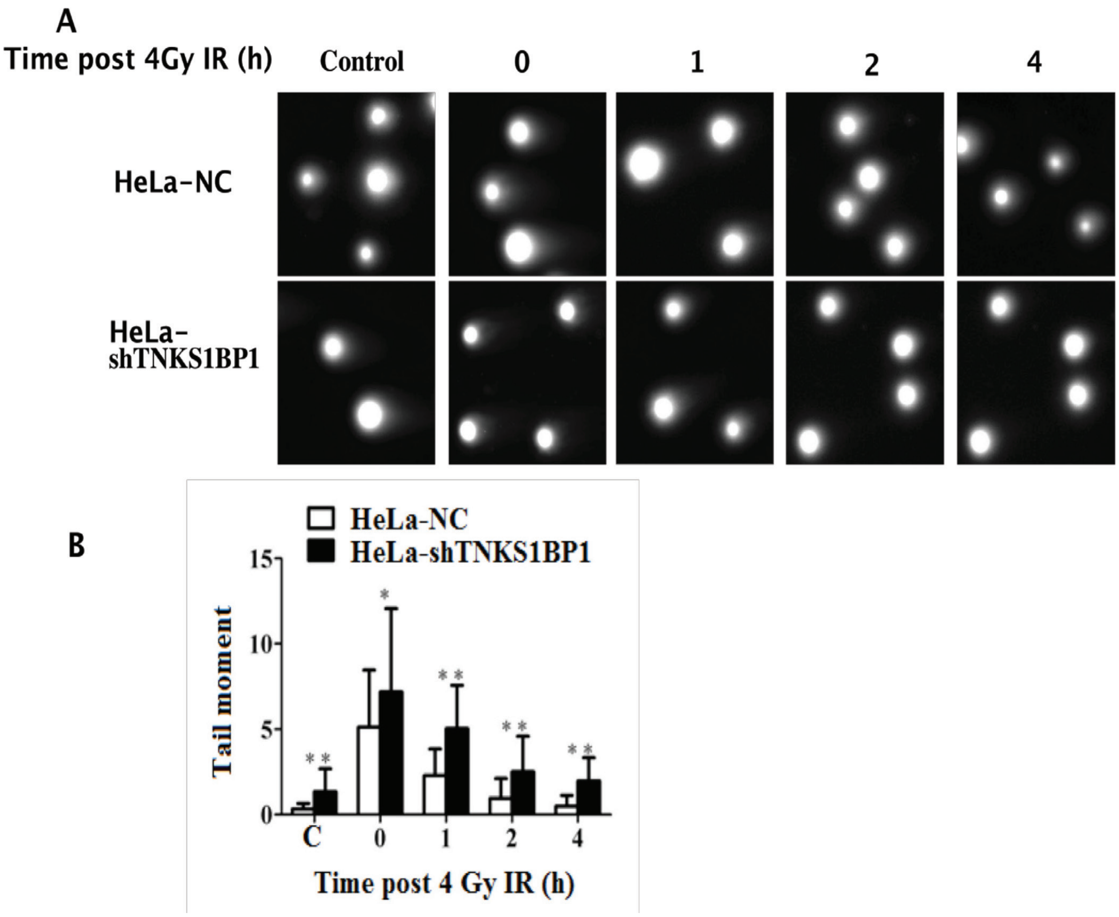

Supplementary Figure 1: Loss of TNKS1BP1 leads to defective DNA double-strand break repair. (A) Neutral comet assay analyzed the DNA double-strand breaks and repair in 4 Gy-irradiated cells. **(B)** Repair kinetics of 4 Gy-induced DSBs detected by comet assay. $*P < 0.05$, $**P < 0.01$, comparison between HeLa-shTNKS1BP1 cells and HeLa-NC cells.

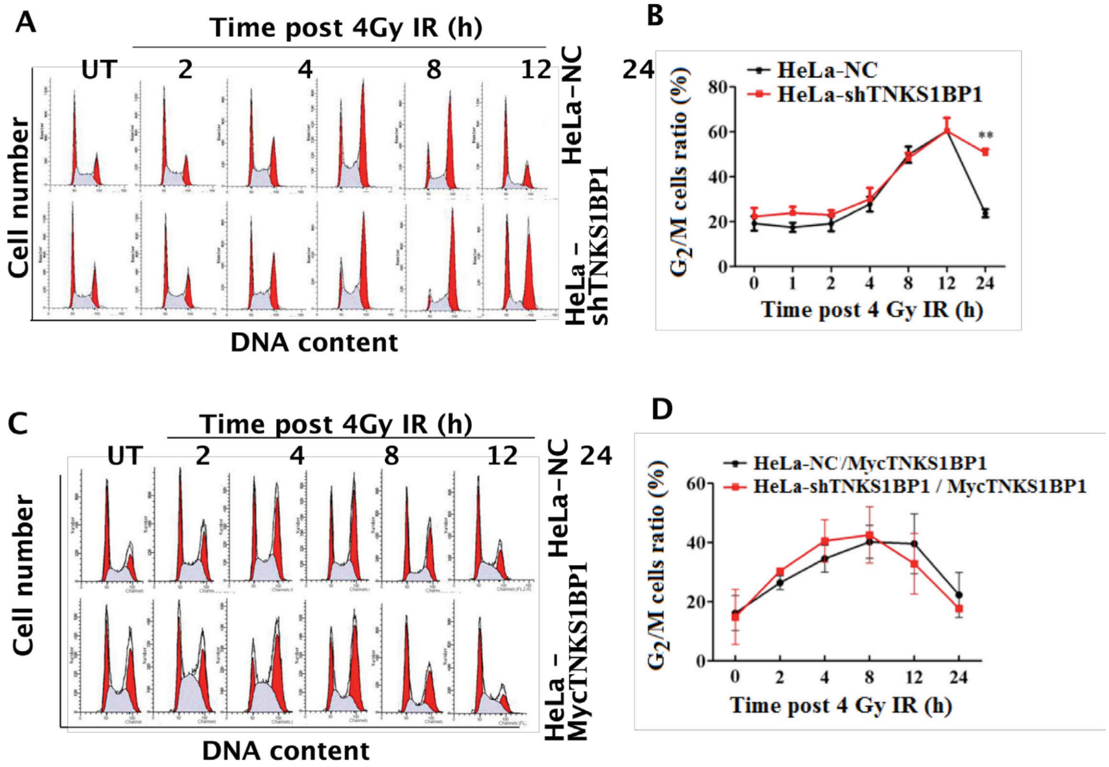

Supplementary Figure 2: Effect of TNKS1BP1 expression on cell cycle progression in response to IR. **(A)** TNKS1BP1 knockdown and control cells were treated with 4 Gy IR and collected at indicated time points postirradiation. **(B)** Quantitative analysis of cell cycle distribution of 4Gy irradiated TNKS1BP1-depleted and control HeLa cells. **(C)** ShRNA-resistant TNKS1BP1 expressing vector was transfected into HeLa-shTNKS1BP1 cells and the cells were treated with 4 Gy and collected at indicated time points postirradiation. **(D)** Quantitative analysis of cell cycle distribution of 4Gy irradiated TNKS1BP1-overexpression and control HeLa cells.
